# Natural and Designed Toxins for Precise Therapy: Modern Approaches in Experimental Oncology

**DOI:** 10.3390/ijms22094975

**Published:** 2021-05-07

**Authors:** Olga Shilova, Elena Shramova, Galina Proshkina, Sergey Deyev

**Affiliations:** 1 Shemyakin and Ovchinnikov Institute of Bioorganic Chemistry RAS, 117997 Moscow, Russia; shramova.e.i@gmail.com (E.S.); galina.proshkina@gmail.com (G.P.); 2 Center of Biomedical Engineering, Sechenov University, 119991 Moscow, Russia; 3 Research Centrum for Oncotheranostics, National Research Tomsk Polytechnic University, 634050 Tomsk, Russia

**Keywords:** targeted toxin, pseudomonas exotoxin, cancer therapy

## Abstract

Cancer cells frequently overexpress specific surface receptors providing tumor growth and survival which can be used for precise therapy. Targeting cancer cell receptors with protein toxins is an attractive approach widely used in contemporary experimental oncology and preclinical studies. Methods of targeted delivery of toxins to cancer cells, different drug carriers based on nanosized materials (liposomes, nanoparticles, polymers), the most promising designed light-activated toxins, as well as mechanisms of the cytotoxic action of the main natural toxins used in modern experimental oncology, are discussed in this review. The prospects of the combined therapy of tumors based on multimodal nanostructures are also discussed.

## 1. Introduction

Cancer treatment has traditionally been based on surgery, radiation, and chemotherapy, which have shown limited therapeutic benefits in patients with metastatic disease. Despite significant advances in the development of systemic treatment, traditional chemotherapeutic agents cause serious side toxicity, restricting treatment to certain therapeutic dosages. In light of this, new approaches to selective treatment are urgently needed.

Protein toxins possessing such features as high cytotoxicity and efficiency have become promising components for anticancer therapy. Cancer cells frequently upregulate surface receptors that promote growth and survival, that is why various antigen-specific proteins including antibodies, antibody fragments (e.g., Fab and scFv), and other protein scaffolds (e.g., affibody and DARPin) have been developed as a moiety to target cancer cells [1,2]. Being genetically encoded, toxins can be expressed as fusion proteins with targeting moieties mentioned above and can have a wide range of modifications to prolong circulation in the bloodstream and increase tumor retention. Complete biodegradation within an organism is also an important advantage of protein toxins as anticancer agents [3,4]. 

Given these advantages, a number of tumor antigen-specific proteins consisting of a targeting domain that recognizes a tumor marker, and a toxic domain based on protein toxin have been developed as potent antitumor agents [4,5,6,7].

In addition to natural protein toxins, designed toxins are also used in experimental oncology, for example, as an alternative to chemical photosensitizers [8,9,10,11]. The main advantage of protein photosensitizers is the opportunity to use a genetic engineering approach to combine cytotoxic and targeting moieties, avoiding chemical conjugation. 

The review discusses the methods of toxins delivery to cancer cells and the compatibility of delivery strategy with mechanisms of protein toxins cytotoxic action.

## 2. Soluble Targeted Toxins

### 2.1. Targeting and Toxic Modules Coupling Strategies

The history of targeted toxins began with the chemical conjugation of natural diphtheria toxin (DT) with anti-lymphocyte antibodies or their F(ab)2 fragments to produce agents for killing lymphoblastoid tumor cells [12]. This strategy helped to couple cell-specific delivery of antibodies with extremely high toxicity of DT, previously shown for mammalian cells [13]. The first generation of immunotoxins used chemical conjugation to couple natural toxins with full-length antibodies [14]. The introduction of hybridoma technology [15] enabled the production of precisely characterized bifunctional agents with a certain specificity. The second generation of immunotoxins arise due to the use of truncated fragments of protein toxins, lacking natural tropism, which helped to reduce *in vivo* side toxicity [16].

Over time, the variety of toxins used in the design of targeted therapy has grown [17,18], but the next breakthrough was made due to molecular cloning, which allowed for the production of the third-generation immunotoxins: fusion proteins consisting of antibody fragments linked to enzymatically active toxin domains [5,19]. Antibodies are mostly used in a single-chain form (scFv); however, over the past 20 years, a variety of nonclassical antibodies have been introduced in biotechnology [1], as well as scaffold proteins of different origin [2,20]. 

Since the development of molecular cloning, the use of protein toxins in recombinant bifuctional and multifunctional proteins has become a straightforward way for targeted agents design. This preference can be explained by the difficulties of precise protein-protein conjugation including the loss of toxin activity or antibody affinity. If we compare the similar targeted toxins reaching the same target we will notice that fused toxins demonstrate higher specific toxicity. For example, we can consider the targeted toxins based on *Pseudomonas aeruginosa* exotoxin A (The 40-, 38-, or 24-kDa portions of the PE without the cell binding domain, are designated as PE40, PE38, and PE24, respectively [21]). The genetically fused 4D5scFv-PE40, containing single HER2-specific trastuzumab variant 4D5scFv as a targeting module, killed HER2-positive cancer cells with IC_50_ value as low as 10–20 pM [22]; at the same time, the IC_50_ value for trastuzumab-PE40 chemical conjugate was about 100 nM [23], although the affinity of trastuzumab alone is higher than that of 4D5scFv: the estimated K_D_ are 1.8 nM and 5.2 nM, respectively [24,25]. Still, the precise chemical conjugation can be achieved by gene engineering. In the recent work a sophisticated modification technology was used. The trastuzumab antibody was engineered to contain unpaired cysteine in the heavy chain, and the unnatural amino acid with an azido group was incorporated into an engineered Pseudomonas exotoxin A (PE24). The two protein molecules were then conjugated site-specifically using a bifunctional linker. The resulting construct demonstrated specific toxicity towards HER2-positive cancer cell in a picomolar range of concentrations [26]. In some cases, the coupling of antibody with a protein toxin can be provided by non-covalent binding of pre-modified modules, for example, with the use of streptavidin and biotin [27]. The proper orientation and stoichiometry can also be provided by design of separate targeting and effector modules, fused to barnase and barstar [28].

### 2.2. Factors Affecting a Targeted Toxin Efficiency

**Figure 1:**
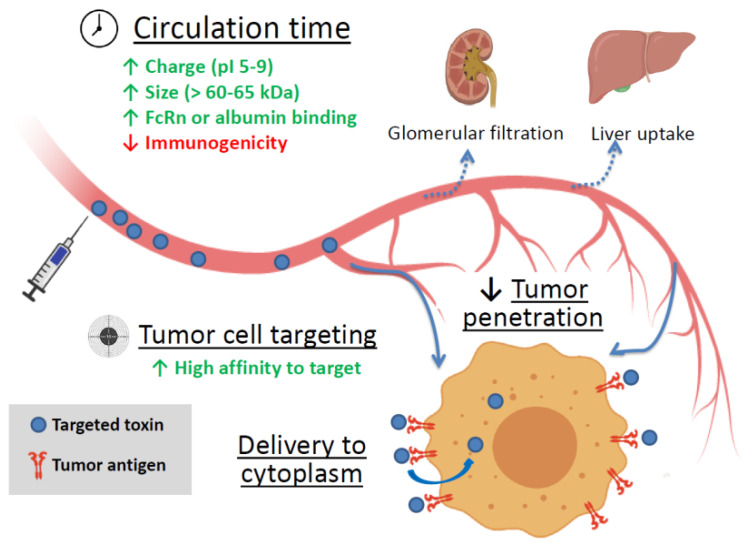
The main factors affecting the efficiency of targeted toxin. Green up arrows—factors enhancing circulation time and tumor cell targeting. Red down arrow—reducing factor. FcRn is the neonatal Fc receptor.

Soluble targeted toxins are thought to be the embodiment of a “magic bullet” idea. Being applied systemically, these agents can reach disseminated, metastatic, or inoperable tumors and kill cancer cells. Still, there are several factors affecting the efficiency of targeted toxins (summarized in Figure 1).

The first and the foremost factor is the agent’s affinity for the tumor antigen. Sometimes the natural tropism of the toxin can be used in cancer therapy, for example, it is possible for the Shiga toxin. Shiga toxin consists of two non-covalently attached parts, the enzymatically active moiety A (StxA) and the non-toxic pentameric binding moiety (StxB) that binds to the glycosphingolipid globotriaosylceramide (Gb3) at the surface of target cells and is then internalized by endocytosis [29]. The expression of Gb3 is relatively restricted in normal human tissues, but it is highly expressed in many types of cancers, including B-cell lymphomas, as well as testicular and colon tumors [30]. StxB was used as a targeting module for fluorescent imaging of human colon cancer cells xenografts in mice, though the accumulation in normal tissues was also considerable [31]. The injections of natural holotoxin were successfully used to kill human cancer cells in murine xenograft models [30]. The anthrax toxin protective antigen (PA) also targets receptors that can be upregulated in tumors, namely tumor endothelial marker 8 (TEM8, ANTXR1) that is involved in tumor angiogenesis [32]. This feature can be used for targeting recombinant toxins to tumors *in vitro* and *in vivo* [33]. Yet, the anthrax toxin PA has another major target, the receptor encoded by capillary morphogenesis gene 2 (CMG2, ANTXR2), that is more widely expressed in normal tissues [34]. To decrease side toxicity the mutated PA variants with predominant binding to TEM8 were obtained [35].

However, the accumulation of most natural protein toxins in the tumor is insignificant, and targeting moieties or tumor-accumulating nanostructures are used to improve drug delivery. If a toxin has natural tropism to surface molecules of human cells, the receptor-binding domains are usually removed. Cancer antigen targeting is usually provided by antibodies, antibody fragments or alternative scaffolds [1,2]. Proper tumor accumulation can be achieved by the selection of targeting molecules with high affinity to the antigen, though the optimal range of affinity can depend on the biology of the target. For example, in case of tumors expressing epithelial cell adhesion molecule (EpCAM), it is better to use antibodies with moderate affinity rather than with high affinity, otherwise the treatment can cause serious side effects [36]. 

Another feature that is important for reaching the tumor is the circulation time. It is affected by several factors, including molecule or complex size, charge (the optimal pI range is 5 to 9 [37]), and immunogenicity [38,39]. The renal filtration cutoff is estimated at 60–65 kDa [40], smaller proteins are cleared quickly and are less likely to reach the target. Increasing the size of the artificial protein or complex can be accompanied by the introduction of multivalency. For example, the use of barnase–barstar modules fused to 4D5scFv made it possible to assemble di- and trimeric complexes with increased avidity and molecular weight (81 kDa and 132 kDa versus 30 kDa of 4D5scFv monomer) [41]. Another strategy involves the use of protein motifs that increase circulation time of a fuse protein. Antibody Fc efficiently recirculates due to neonatal receptor FcRn [42] and introduction of Fc into fuse protein can increase the circulation time of a construct. A similar effect can be achieved by the use of serum albumin or albumin-binding proteins in a fuse construct [42,43,44]. 

The immunogenicity of a toxin is a complex characteristic that usually decreases the circulation time. On the one hand, molecules that are efficiently recognized by macrophages of reticuloendothelial system are rapidly cleared from blood. On the other hand, the subsequent presentation leads to the production of toxin-specific antibodies, which limits the toxin efficiency in case of repeated treatment. The reduction in immunogenicity can be reached either by gene engineering or by chemical modification of fusion toxins, such as PEGylation or the removal of compounds recognized by macrophages [45]. For ricin it was shown that oligosaccharides facilitate the toxin uptake by macrophages through binding to CD206 mannose receptor [38,46], which reduces circulation time and may contribute to successful protein presentation. Ricin oligosaccharides were also shown to interact with glycosylated IgA and IgM [47], and this can also contribute to toxin clearance from circulation and better presentation due to enhanced macrophage uptake of the immune complexes. In case of ricin the circulation time can be increased by chemical deglycosylation of the toxin [39,48]. As for the protein part of a toxin, it can be modified for worse recognition and activation of immune cells through the gene engineering. This strategy was successfully used for modifying DT and PE. To reduce the immunogenicity of DT seven point mutations were introduced to the surface highly hydrophilic amino acids that were located away from the catalytic site according to the X-ray structure. The resulting modified truncated diphtheria toxin triggered the production of lower levels of antibodies comparing to non-modified protein in mice without losing more than a log of activity [49]. In case of PE the more sophisticated method was used. B-cell epitopes were identified by using a panel of antibodies derived from immunized mice and the human antibodies present in the sera of patients treated with PE38-based recombinant immunotoxins (IT) [50]. The exact location of the epitopes was determined by introducing individual alanine replacement of bulky amino acids and subsequent loss of binding analyzing a panel of monoclonal antibodies [51]. The PE immunogenicity was further reduced by removing a large part of PE38 domain II [52]. Furthermore, an immunodominant T-cell epitope in PE-based recombinant ITs was identified and eliminated. This was achieved by incubation of peripheral blood mononuclear cells with a toxin to stimulate T-cell activation, subsequent re-stimulation to overlapping peptides derived from PE38, and quantitation of the responses in an IL2-enzyme-linked immunospot assay. The low immunogenic toxin has good cytotoxic and anti-tumor activity towards human cell lines, patient-derived cells, and mouse tumor models [53].

Sustained circulation is important because it provides an efficient accumulation of a targeted toxin in the tumor. In many cases penetration into a solid tumor is facilitated due to malformation of the capillary network. This phenomenon is called the enhanced permeability and retention (EPR) effect [54]. However, this effect is not always sufficient to ensure a required drug penetration, which is impeded by intercellular junctions of both endothelial and cancer cells. There are a number of other factors preventing proper drug penetration into a tumor, including tumor stroma that provides physical barriers for therapeutic agents and a poor vascularization of the tumor. Furthermore, the lymphatic network is rather weak in solid tumors, and an enhanced permeability of blood vessels together with proliferation of cancer cells leads to an increased intratumoral pressure [55]. Still, nowadays a number of virus protein-based strategies for enhancing the intratumoral diffusion exist, including cell junction targeting and induction of temporal epithelial-to-mesenchymal transition [55,56]. It was also demonstrated that botulinum neurotoxin (BoNT) briefly opens tumor vessels, allowing more effective destruction of cancer cells by radiotherapy and chemotherapy [57,58], but the possible benefit of tumor treatment with botulinum toxin in complex with other protein toxins is yet to be investigated.

The next step is to provide a contact of an effector module with its target cellular compartment in a tumor cell. For photosensitizers plasma membrane itself can serve as a target, and in this case a delivery of an agent to a cancer cell surface marker is sufficient to kill the cell [10]. In this case the targeting module should have high affinity to a target receptor and its interaction with the tumor antigen should not decrease the phototoxin efficiency [59,60,61]. 

Nevertheless, if an effector module needs to interact with cytoplasmic or nuclear compounds, its internalization and intercellular transport is required for efficient work. The most dangerous toxins have evolved to cheat cell trafficking systems or cross cell membrane, and the targeted agents based on these toxins can cope with cytoplasm delivery themselves [7]. For some proteins, mainly DT, PE, Stx, and ribosome inactivating toxins, the intercellular trafficking is well studied [30,62,63]. In some cases, parts of these toxins responsible for cytoplasm delivery are precisely mapped and can be used for improvement of the endosome escape efficacy of other therapeutic agents. For example, StxB can be used as a tool for cell delivery of various cargo through endocytosis and retrograde traffic [64]. In turn, the translocation domain of PE was used to enhance cytoplasm delivery of hybrid agents based on Shiga-like toxin 2; the resulting fusion protein N8A-TDP-Stx2B inhibited the growth of hepatocellular carcinoma cells HepG2 with a half-maximal inhibitory concentration (IC_50_) of approximately 1 nM and was further tested in mouse xenograft model [65]. For other protein types the introduction of cell-penetrating peptides into fuse protein was proven to be useful. These short 30–35 amino acid peptides, mainly HIV-derived TAT, Drosophila’s penetratin, and VP22 from Herpes simplex virus [66] can be easily introduced into fuse proteins and enhance their delivery into the cytoplasm [66,67]. Pore-forming proteins can also enhance penetration into tumor cells and were successfully used in dual targeting strategy to improve cytoplasmic delivery of the type I ribosome-inactivating toxin Gelonin [68]. 

## 3. Targeted Toxins as Components of Nanoagents

Despite the successful use of immunotoxins, immunotherapy strategies are still expensive, mainly due to the complicated preparation process. Immunotoxins can also stimulate the host immune system and trigger the production of neutralizing antibodies. Intravenous administration of targeted protein toxins may be characterized by poor pharmacokinetic profiles in addition to non-specific distribution in tissues and organs of the body and can cause serious side effects including systemic toxicity. Besides, the penetration of anticancer drugs into tumor tissues is usually low and the high doses of drugs are required for treatment [69,70]. The use of nanocarriers, especially the targeted ones, for delivering toxins to tumor foci may improve the pharmacokinetics and pharmacodynamics of agents, control drug release, improve the specificity, increase internalization and intracellular delivery, and reduce systemic toxicity [71]. Nanocarriers can facilitate selective accumulation in tumors via the enhanced permeability and retention (EPR) effect and active cellular uptake [72]. Among various nanoscale drug carriers, liposomes, polymeric nanoparticles and noble metal nanoparticles have demonstrated the greatest potential in clinical application [73,74,75]. 

The nanocarrier size should be somewhere between 10 and 100 nm for efficient extravasation from the fenestrations in leaky vasculature and for the avoidance of the filtration by the kidneys and the unspecific capture by the liver. The charge of the particles should be neutral or anionic for efficient evasion of the renal elimination. Besides, the nanocarriers should be hidden from the reticuloendothelial system (RES), which destroys any foreign material through opsonization followed by phagocytosis [76]. Recent works on reversible RES blockade either by nanoparticles or by opsonized red blood cells provides additional strategies for prolongation of circulation [77,78].

Liposomes are closed spherical vesicles formed by one or several phospholipid bilayers surrounding an aqueous core, in which hydrophilic drugs can be entrapped. Numerous factors define liposome properties, such as lipid composition, a number of lipid bilayers, size, surface charge, and the method of preparation [79]. They can be also coated with inert and biocompatible hydrophilic polymers, such as polyethylene glycol (PEG), to avoid rapid elimination from the systemic circulation by the RES after opsonization with serum proteins and grafted with targeting ligands [76]. 

Pilot studies on liposomal delivery of toxins to cancer cells *in vitro* were published back in the early 80s of the last century. In 1982, McIntosh and Heath studied the cytotoxic effect of Gelonin, a potent inhibitor of protein synthesis from *Gelonium multiflorum*, delivered to different tumor and normal cell lines using liposomes of various compositions [80]. Jansons and Panzner in 1983 managed to carry out passive liposomal delivery of fragment A of diphtheria toxin (DTA) without losing its cytotoxic properties [81]. To enhance target cell specificity, Collins and Huang have proposed pH-sensitive immunoliposomes coated with fatty acid-derivatized antibody against the mouse major histocompatibility antigen H-2K^k^ for targeted delivery of a DTA to free toxin-resistant murine cells and demonstrated its high cytotoxicity [82]. Later the targeted delivery of DTA via tumor-specific immunoliposomes and high anti-tumor activity on human ovarian carcinoma cells even in the presence of neutralizing anti-diphtheria toxin antibodies was demonstrated [83]. Circulating neutralizing anti-toxin antibodies protect against non-specific action of toxin and considerably limit the therapeutic use of immunotoxins due to early inactivation and, in particular, in case of multiple injection schemes [84]. 

The toxin delivery system based on pH-sensitive non-targeted liposomes simultaneously loaded with a pore-forming protein listeriolysin O and Gelonin, was quite effective [85]. Listeriolysin O mediated escape of the toxin molecules from the endosome into the cytosol after liposome internalization. Proteoliposomes killed B16 melanoma cells in vitro with a Gelonin IC_50_ in subnanomolar range. The treatment by direct intratumor injection into subcutaneous solid tumors of B16 melanoma in a mouse model showed that the proposed pH-sensitive liposomes were more effective in curtailing tumor growth rates than control ones.

Liposomes have proven to be an efficient vehicle for delivering a high molecular weight neurotoxin botulinum toxin A to treat hypersensitive bladder and overactive bladder (OAB) without systemic injection [86]. Intravesical lipotoxin administration cleaved SNAP-25, inhibited calcitonin gene-related peptide release from afferent nerve terminals, and blocked rat bladder hyperactivity induced by acetic acid [87]. Besides, intravesical lipotoxin instillation effectively reduced frequency episodes 1 month after treatment in OAB patients without any increase in postvoid residual or the risk of urinary tract infection [88].

Yaghini and colleagues proposed the use of liposomes for passive simultaneous delivery of protein toxin saporin and photosensitizer disulfonated tetraphenylporphine for light-triggered cytosolic release [89]. They showed that liposomes loaded with saporin and functionalized with cell penetrating peptides (Tat 48-57, cell-permeable peptide, derived from HIV-1 transactivator of transcription (Tat) protein residue 48-57), some of which are connected via a flexible linker with photosensitizers, effectively bind to and are internalized into tumor cells *in vitro*. When exposed to light, ROS-mediated damage of internalized liposomes was induced, toxin molecules were released into the cytosol and cytotoxicity of saporin was significantly enhanced in comparison with the effect of free toxin exposure. The two-modal photodynamic and cytotoxic effects of the described proteoliposomal system led to almost 100% death of the irradiated cells at nanomolar concentrations of saporin with short exposure times.

The potential of thermosensitive liposomes as nanocarriers for high-molecular weight cytotoxins have been recently demonstrated [90]. The presence of 10% mol 1-stearoyl-2-hydroxy-sn-glycero-3-phosphatidylcholine (MSPC) in the liposomes provided them with a homogeneous size, a suitable temperature range for hyperthermia, and effective release of their cargo after heating. *In vitro* experiments with mouse CT26 colon carcinoma cells confirmed that proteoliposomes contained a ribosome-inactivating protein toxin Mistletoe lectin-1 (ML1), strongly inhibited tumor cell viability upon mild hyperthermia treatment, and this approach represents a promising strategy for local passive tumor delivery for macromolecular cytotoxins.

Gao and co-authors developed the PEGylated immunoliposomes conjugated with anti-HER2 Fab and loaded with PE38KDEL for targeted delivery of protein toxin to HER2-positive cells [91]. PE38KDEL is a 38 kDa mutant form of PE and exhibits superior anti-tumor activity and low non-specific toxicity [92]. The immunoliposomes were less than 200 nm in diameter, had a high drug loading capacity and antibody conjugation efficiency and could be efficiently bound to and were internalized into HER2-overexpressing breast cancer cells, resulting in potent cytotoxicity *in vitro* in a picomolar concentration of toxin. It is worth noting that targeted immunoliposomes were more cytotoxic than non-targeted ones in HER2-overexpressing tumor cells. 

**Figure 2:**
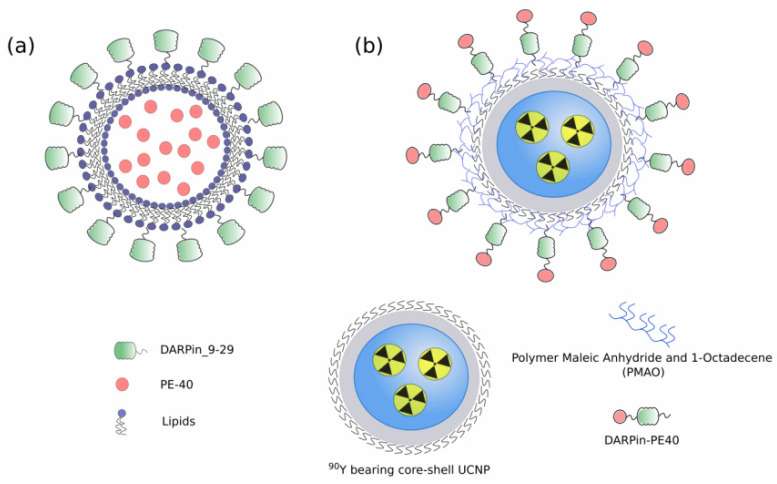
(**a**) Small unilamellar targeted liposomes containing PE40 [93]; (**b**) Hybrid biofunctional nanocomplex based on radioactive 90Y bearing core-shell UCNP and functionalized with targeted toxin DARPin-PE40 [97].

Recently, a new method has been proposed for the preparation of small (80–90 nm) unilamellar antigen-targeted liposomes containing large amounts (thousands of protein molecules per liposome) of highly toxic PE40 [93] (Figure 2a). Efficient encapsulation of the proteins was achieved through electrostatic interaction between positively charged toxin proteins at pH lower than pI and negatively charged liposome membrane. The external surface of proteoliposomes were functionalized with covalently coupled DARPin_9-29 using “click chemistry” through a relatively long flexible linker. Functionalized proteoliposomes specifically bind to HER2-positive cells and after internalization cause cell death at subnanomolar concentrations [94].

Furthermore, this method was used for obtaining DARPin_9-29 functionalized liposomes loaded with ribonuclease barnase [95,96]. Targeted liposomes loaded with barnase effectively inhibit the viability of HER2-positive cells, and the severity of the cytotoxic effect correlates with the expression level of the HER2 receptor.

Nanoparticles (NP) have considerable potential for cancer imaging and therapy due to their small size and prolonged circulation. In addition, synthesis and formulation of NPs are simple and cost-effective, and because of their small size, NPs are not antigenic or immunogenic themselves. Polymeric NPs also turned out to be very promising as effective nanocarriers of protein toxins. Chen and co-authors proposed to use poly(lactic-co-glycolic acid) (PLGA) biodegradable and biocompatible polymeric NPs to targeted delivery of PE38KDEL to cancer cells [98]. PE38KDEL-loaded PLGA NPs were functionalized using Fab’ fragments of a humanized anti-HER2 monoclonal antibody to target the HER2 antigen. In vitro experiments demonstrated the specific high-affinity binding of PE38KDEL-loaded PLGA Nps to breast cancer cells overexpressing HER2; the antitumor activity of PE38KDEL-loaded PLGA Nps was higher and nonspecific toxicity was lower than that of free PE38KDEL immunotoxin. In the developed xenograft model of HER2-overexpressing tumor, administration of immunonanoparticles showed a much better therapeutic efficacy in inhibiting tumor growth and reducing systemic toxicity of PLGA NPs as compared with free immunotoxin. 

In another work [99], silver nanoparticles (Ag NP) for PE38 non-targeted delivery to human adenocarcinoma cells in vitro were proposed. The Ag NPs loaded with toxin demonstrated a severe cytotoxic effect on the proliferation of the breast cancer cells and the P53-dependent apoptosis mitochondrial pathway was the major pathway of cell death induced by this nanotoxin.

A successful use of gold nanoparticles (GNP) as carriers of protein toxins has been demonstrated in the work by Bhowmik et al. [100]. They showed that PEGylated gold nanoparticles conjugated with Naja Kaouthia Cytotoxin 1 (NKCT1), a protein toxin from the Indian cobra (*Naja kaouthia*) venom, work synergistically and lower the application dose and duration of action for NKCT1, ensuring that NKCT1 is released by GNPs into the target cells in a controlled manner, the cytotoxic effect of NKCT1 is two to threefold stronger and its side toxic effects are minimized as compared to the unconjugated NKCT1. These conjugated GNPs-NKCT1 exhibited high anti-leukemic activity *in vitro,* induced cell cycle arrest, and promoted apoptosis-regulating activities, such as nuclear fragmentation. It was later shown that GNPs-NKCT1 inhibits growth of different cancer cell lines, and that in the case of normal cell lines treated with GNPs-NKCT1, cell death was significantly less than in the treated cancer cells [101]. In immunocompetent mice with liver tumors induced by diethylnitrosamine (DEN) injection, the number and size of tumors were much smaller in mice treated with GNPs-NKCT1 than in mice treated with NKCT1, and were comparable to the results of 5-F fluorouracil therapy.

It is known that combining multiple synergistic therapeutics may reduce the dosage requirements and be beneficial in cases of tumors resistant to a single drug therapy. It was realized that the best outcome is achievable when both toxic agents enter target cancer cells at the same time in the form of a single supramolecular structure or two agents are targeted at different sites of the same oncomarker on the target cell but do not compete for binding. To implement the first approach in combined radio- and chemotherapy, up-conversion nanoparticles (UCNP) coupled to two therapeutic agents were proposed: beta-emitting radionuclide yttrium-90 (^90^Y) fractionally substituting yttrium in UCNP, and a fragment of the exotoxin A derived from *Pseudomonas aeruginosa* genetically fused with a targeting DARPin specific to HER2 receptors (Figure 2b) [97]. The synergistic effect of multifunctional nanocomplexes is markedly enhanced in comparison with monotherapy carried out separately *in vivo*, which allows reducing the concentration of both toxic agents (radioisotope and immunotoxin) by about 2200 times. The photophysical properties of UCNPs made it possible to perform background-free imaging of the distribution of therapeutic nanoparticles in the body and non-invasively record the response to treatment in real time.

Another work proposed a new cancer therapy strategy that selectively targets two different binding sites for HER2 with therapeutic compounds, which act through completely different mechanisms of action, for bimodal chemotherapy and immunotherapy [102]. PLGA NPs functionalized with affibody Z HER2:_342_ for targeting subdomain III and IV of HER2 and immunotoxin containing a low-immunogenic modification of PE (LoPE) and DARPin_9-29 for targeting subdomain I of HER2 were used. PLGA NPs were loaded with an imaging fluorescent dye Nile Red and a chemotherapeutic drug doxorubicin. The proposed dual targeting strategy can drastically enhance anticancer therapy of HER2-positive cells, which made possible a 1000-fold decrease in the effective drug concentration in vitro and a significant enhancement of HER2 cancer therapy compared to monotherapy *in vivo*. 

In support of the new nanomedical concept of self-assembling self-delivered drugs that act in the absence of any external vehicle, self-assembling toxin-based nanoparticles were designed [103]. Conveniently engineered, the protein toxins, namely segments of the diphtheria toxin and the *Pseudomonas aeruginosa* exotoxin, targeted to CXCR4+ cancer stem cells, have been successfully produced and purified in bacteria. Protein toxins self-organized as toroid nanoparticles of 30–90 nm. In this form, they penetrated into CXCR4+ target cells and promoted receptor specific cell killing both in vitro and *in vivo*, playing a dual role as a drug and a carrier, and causing programmed cell death and destruction of tumoral tissue after administration of a single dose. The systemic administration of both nanostructured drugs in a mouse xenograft model of colorectal cancer promoted efficient and specific local destruction of the tumor tissues and a significant reduction in the tumor volume. The developed self-assembling system of toxin-based protein NPs has subsequently proven itself well in the treatment of colorectal cancer [104] and diffuse large B-cell lymphoma [105].

## 4. Cytotoxic Mechanisms of Natural Toxins

The killing mechanisms of protein toxins can vary, but they differ from the mechanisms that are implemented in conventional chemotherapy [4], so an obtained resistance to chemotherapeutic agents does not affect the effectiveness of protein toxins. Furthermore, the mechanism complementation can provide a synergistic effect of combined therapy. In addition, protein toxins are not mutagens and should not accelerate tumor progression due to enhanced mutagenesis. They can be mass-produced cheaply in bacteria as homogeneous proteins [5]. 

Toxins of bacterial and plant origin commonly used as cytotoxic component in chimeric proteins in anticancer therapy are summarized in Table 1. The most toxic proteins include enzymes that inhibit translation at the elongation step. Unsurprisingly, most of them arise from natural toxins that have been effectively preselected by evolution. 

### 4.1. Toxins Inhibiting Protein Synthesis

Enzymes such as diphtheria toxin (DT), pseudomonas exotoxin A (PE), ricin, Shiga toxin (Stx), abrin, and similar agents can be considered as the most versatile toxic modules, as they exhibit not only toxic properties, but also the ability to penetrate into a target cell and reach its cytoplasm [62,111,113]. These features make natural toxins compatible with most delivery strategies used in experimental cancer therapies. On the other hand, these protein toxins have some disadvantages that should be considered developing therapy, mainly the side effects including vascular leak syndrome, hepatotoxicity, and kidney damage [84,121,122]. In addition, Shiga toxin is notorious for its ability to cause hemolytic uremic syndrome (HUS), potentially leading to life-threatening complications [123,124]. Bacterial and plant toxins in their natural forms can also show high immunogenicity, which limits the safety and effectiveness of therapy. Nevertheless, all these issues can be solved with the help of contemporary modification techniques and toxin delivery methods, which we are discussing later.

Another promising class of protein toxins are ribonucleases like barnase [112,113] and binase [114]. Cleavage of messenger RNA is a universal mechanism of cell killing, as any human cell depends on protein synthesis. These enzymes should be most active when delivered to the cytoplasm, but experimental data demonstrate that barnase in the form of a targeted recombinant protein that binds to the surface HER2 receptor enters the cell via receptor-mediated endocytosis and can induce apoptosis in cancer cells [112,113], although the mechanism of its escape from endosome remains unclear. Similar results were obtained for conjugates of mammalian RNAse A with antibodies to transferrin receptor or CD5 tested on cancer cells expressing respective target molecules [125]. Cytotoxic activity was even shown for untargeted ribonucleases, namely RNAse A, and its homolog onconase, which are likely to be transported to cancer cells in a non-specific manner [126]. Thus, at least some RNAses are capable of crossing cytoplasmic or vesicular membrane and reach cytosol, so these agents are also compatible with a variety of delivery techniques.

### 4.2. Toxins Disrupting Cell Signaling

Anthrax toxin (AT) causes cell death due to disruption of kinase signaling and uncontrolled generation of cAMP. It consists of three proteins: protective antigen (PA, 83 kDa), lethal factor (LF, 90 kDa) and edema factor (EF, 89 kDa) that are non-toxic alone in mouse models, but form active bipartite combinations. The combination of LF and PA generates lethal toxin (LT), while EF combined with PA comprises edema toxin (ET) [115,127]. PA binds to either of the two known natural receptors, tumor endothelial marker 8 (TEM8 or ANTXR1) or capillary morphogenesis gene 2 (CMG2 or ANTXR2). Upon binding to cell surface PA is cleaved by furin protease and its C-terminal 63-kD moiety (PA63) can form heptamer or octamer [128]. The oligomerization of PA provides the binding site for LF or EF and also triggers internalization of the toxin complex via a lipid raft-dependent clathrin-mediated process [129]. In the endosome acidic environment PA oligomer inserts into lipid bilayer forming cation-selective pore that also provides the translocation of the unfolded LF and EF into cytosol [130]. 

Once in the cytoplasm, EF acts as a calmodulin-dependent adenylate cyclase which increases the cAMP concentration in cells [131]. LF is a zinc metalloproteinase that cleaves mitogen-activated protein kinase kinases (MAPKK) in their N-terminal regions and Nlrp1. The cleavage of MAPKKs disrupts several signaling pathways, including the ERK1/2, JNK/SAPK, and p38 pathways, which are important for cell survival, proliferation and cell cycle regulation [132]. The cleavage of Nlrp1 by LT causes toxin-induced inflammasome activation and IL-1β release [133].

The three components of AT are individually non-toxic, and the PA component must be proteolytically activated prior to cell intake. These unique features render anthrax toxin attractive for tumor therapy. As it was already mentioned in Section 2.2 the PA target TEM8, can be upregulated in several types of tumors, [32]. This feature can be used for targeting recombinant toxins to tumors in vitro and *in vivo*. A number of melanoma cell lines are sensitive to LF, especially those bearing the activating V600E B-RAF mutation [134,135]. This selective toxicity was also observed *in vivo*: intraperitoneal injections of LT caused partial and complete regressions of subcutaneous tumor xenografts [136]. The selectivity of the toxin was further improved by replacing the original furin cleavage site by matrix metalloproteinase (MMP) cleavage site. The resulting MMP-activated PA with LF efficiently treated melanoma xenografts, and lung and colon carcinoma xenografts irrespective of the B-RAF status [137]. The idea of retargeting was also used in MMP- or urokinase plasminogen activator (uPa)-activated PA in combination with FP59 toxin consisting of anthrax toxin lethal factor residues 1-254 fused to the ADP-ribosylation domain of Pseudomonas exotoxin A [138,139]. This approach was further improved by engineering PA variants that can only form octamers after activation by both of the tumor-selective proteases, uPa and MMPs and, thus, achieved a safe dual-activity dependent delivery system [140]. 

The use of different toxins fused to LF has proven to be useful: many types of tumors are not sensitive to MAPK signaling inhibition and LT itself is not efficient, but LF can provide targeting and cytoplasmic delivery of other protein toxins, such as tetanus toxin [141], pseudomonas exotoxin A [138,139,142], diphtheria toxin A [143,144], and Shiga toxin [144] (summarized in [115]). 

Retargeting of PA with the help of fused proteins can also be achieved. An elegant approach based on PA–LF complex formation was published in 2012 [145]. The authors used a mutant PA that is unable to bind either TEM8 or CMG2 due to two point mutations, N682A and D683A. This mutant PA (mPA) was fused C-terminally to human epidermal growth factor (mPA-EGF). Working as a pre-targeting module mPA-EGF bound to EGFR-positive cells and, in turn, attracted LFn-DTA protein (LF fused to receptor-binding domain of diphtheria toxin) to the cells. The use of mPA-EGF/LFn-DTA combination resulted in high protein synthesis inhibition (IC_50_ 0.01 nM LFn-DTA) on epidermal growth factor receptor-positive human A431 tumor cells while protein synthesis in receptor-negative CHO cells was not affected at concentrations of up to 10 nM LFn-DTA. mPA was also used as an effector module in fuse with HER2-specific affibody ZHER2 [146]. The resulting mPA-ZHER2 protein was combined with either LFn-DTA or LFn-RTA which resulted in strong inhibition of protein synthesis and high cytotoxicities on HER2-positive cells, while HER2-negative cells were not affected.

To sum up, we can conclude that anthrax toxin provides a variety of tools for tumor targeting both through the natural tropism and toxicity and through the ability to translocate to cytoplasm thus delivering other toxins to cancer cells. To date, the dual-targeting strategies involving cell surface receptor recognition and tumor-specific activation look the most promising.

### 4.3. Proteins Inducing Oxidative Stress

Another intriguing application of protein toxins is their use as photosensitizers (molecules capable for reactive oxygen species production (ROS) upon irradiation) in deep-penetrating photodynamic therapy (PDT). 

**Figure 3:**
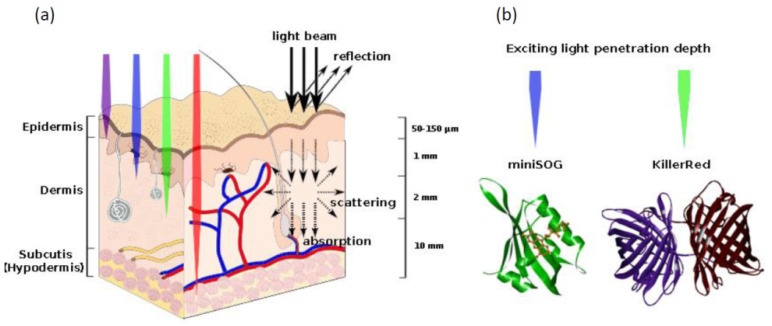
(**a**) Light propagation through the tissues; (**b**) Genetically encoded PSs. 3D model (ribbon representation) of miniSOG (PDB entry 6GPV) and KillerRed (PDB entry 2WIQ) was made using DS ViewerPro 5.0 software.

Photodynamic therapy (PDT) has been considered as a potential therapeutic intervention against diseases due to its minimally invasive nature, localized therapy with minimal or no damage to healthy tissues, and fast healing process [147,148,149]. In PDT, three elements are required simultaneously: a light-activated photosensitizer (PS), a light source with an appropriate wavelength, and surrounding oxygen [150]. When illuminated by light at a specific wavelength, PS absorbs the light energy and can be promoted into an excited singlet state. The energy of the excited singlet state can be dissipated either by thermal decay, or the emission of fluorescence, or moving to a lower energy excited triplet state via intersystem crossing. At the excited triplet state, the PS can undergo a photochemical reaction with the surrounding molecules to generate reactive oxygen species, such as superoxide anion, hydroxyl radical, hydrogen peroxide, or singlet oxygen [151]. As ROS have a short range of action and a short lifetime, the primary targets of photodamage are molecules and cells that are proximal to the area of ROS production after irradiation. Thus, PDT is a minimally invasive technique that allows specific and localized therapeutic effects on cancer cells. However, the need of external light source for PS activation hinders the application of PDT for deep-seated neoplasm due to the limited penetration depth of the external light in biological tissue [150] (Figure 3a).

Fluorescent proteins capable of reactive oxygen production (ROS), a new type of biological photosensitizers, are considered to be a promising substitute for current synthetic photosensitizes used in photodynamic therapy (PDT).

There are only two genetically encoded PSs reported so far: dimeric GFP-like far-red fluorescent protein KillerRed [8] (with its monomeric version SuperNova [152] and green fluorescent flavoprotein miniSOG [153] (Figure 3b). The main mechanism of KillerRed phototoxicity includes free radical formation (mainly O_2_^−^) through one electron reduction in O_2_. Mini-SOG is capable of producing primarily but not exclusively O_2_^−^ [6,154,155].

This new type of biological PSs is considered as a promising substitute for current synthetic photosensitizes used in PDT, and as it was shown in a series of in vitro investigations, miniSOG and KillerRed possess phototoxicity equal or exceeding that of commonly used PSs or other fluorescent chromoproteins [8,9,156]. Remarkable phototoxicity, in addition to water solubility and biocompatibility, has placed genetically encoded PSs among the top ideal hydrophilic candidates for PDT, which has been successfully proven for photoablation in cell models [9,157,158,159,160].

Although genetically encoded PSs exhibited high phototoxicity in cultured tumor cells in vitro, as well as in transparent animals [161,162,163,164], the achievement of substantial photoablation effect in a tumor xenograft in in vivo model is a complicated task [157]. For example, Ryumina and coworkers have shown that miniSOG, a 106 amino acid green fluorescent flavoprotein generated from Arabidopsis phototropin, stably expressing in xenograft tumor model, does not cause substantial photoablation of the tumor under irradiation due to limited penetration of excitation light deep into tissues [157]. 

**Figure 4:**
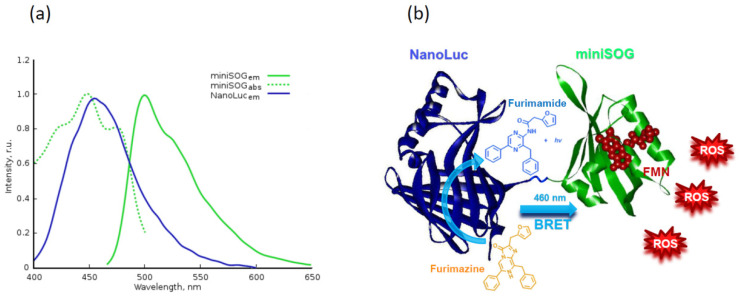
Bioluminescence system based on luciferase, furimazine, and miniSOG. (**a**) Normalized emission spectrum of furimamide (NanoLuc_em_) and normalized absorption (miniSOG_abs_) and emission (miniSOG_em_) spectra of miniSOG. (**b**) Scheme of BRET-mediated system for deep PDT [165,166].

To overcome this challenge, a new elegant approach based on non-radiative energy transfer from donor luciferase-substrate reaction to the acceptor-fluorophore was proposed [165,166]. miniSOG and NanoLuc form a good BRET (bioluminescence resonance energy transfer) pair, in which the emission peak of NanoLuc (in the presence of its specific substrate furimazine) at 460 nm is well matched with the absorption peak of miniSOG at 448 nm (Figure 4) [165]. Using in one genetic construct, the genes encoding phototoxic protein miniSOG (as a PS [153]) and NanoLuc luciferase (as a light source [167]) it was shown that the NanoLuc-miniSOG system is an efficient tool for PDT therapy, where NanoLuc serves as a deep tissue flashlight in the absence of external physical stimuli and chemical co-factors. This system was comprehensively characterized in vitro and it was shown that the intensity of the light emitting by NanoLuc-furimazine bioluminescence system is sufficient to activate miniSOG leading to ROS production in cancer cells, and the photodynamic effect caused by BRET-induced PDT is comparable with that of light-induced PDT [166]. In vivo experiments on animals with xenograft tumors stably expressing NanoLuc-miniSOG gene and treated with luciferase substrate showed apparent tumor growth inhibition. On day 25 after treatment, the tumor volumes in the control groups were increased approximately sixfold, while in the PDT group tumor growth was strongly inhibited, with TGI (tumor growth inhibition coefficient) equal to 71% [168]. To date, this is the only fully genetically encoded system based on bioluminescence resonance energy transfer for PDT *in vivo,* which opens up new prospects for the application of PDT in model organism, regardless of the depth of the tumor.

**Figure 5:**
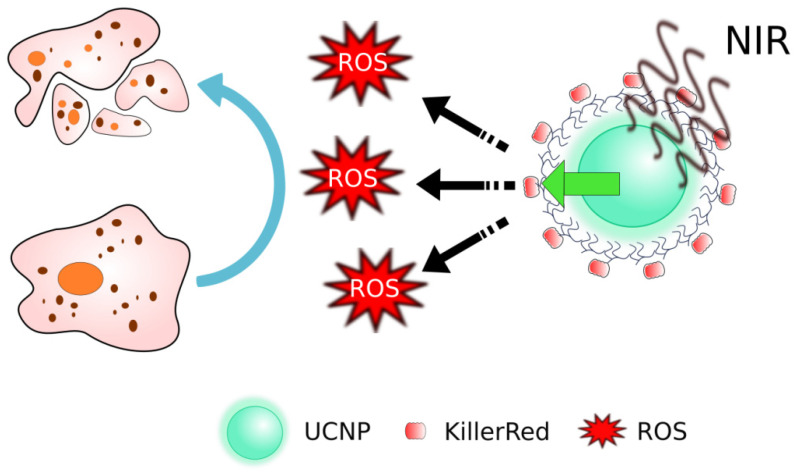
Nanoagents based on KillerRed and upconversion nanoparticls (UCNP). The deep-penetrating near infrared (NIR) light is converted to yellow light that is able to excite KillerRed [116].

Another known to date genetically encoded PS capable of ROS production under exposure to visible light is KillerRed (Figure 3a). When irradiated with yellow-orange light (~582 nm), KillerRed demonstrated efficient production of ROS, the phototoxicity of which was at least 1000 times higher than that of other fluorescent chromoproteins [8,156]. To overcome the shallow penetration depth of excitation light and make possible to use KillerRed in deep-seated tumors, the photosensitizing bio-nanohybrids based on KillerRed and upconversion nanoparticls (UCNP) have been developed (Figure 5) [116]. UCNPs used in this work are able to convert deep-penetrating near infrared (NIR) light to yellow light to excite KillerRed locally. It was shown that being excited by UPNPs, KillerRed efficiently generates ROS that cause cancer cells killing. The KillerRed-UCNPs exhibit excellent colloidal stability in biological buffers and low cytotoxicity in the dark. Cross-comparison between the conventional KillerRed and UCNP-mediated KillerRed PDT demonstrated superiority of KillerRed-UCNPs photosensitizing by NIR irradiation, manifested by the fact that about 70% PDT efficacy was achieved at 1-cm tissue depth, whereas that of the conventional KillerRed dropped to about 7%. Bio-nanohybrids proposed in this work prompts investigation of phototoxic potential of proteins in the visible and even ultraviolet spectral ranges towards their potential utilization in PDT.

### 4.4. Direct Apoptosis Induction

The human proteins that work as apoptosis inducers can also be used as effector modules for cancer therapy. Granzyme B (GzmB) is secreted by cytotoxic T cells and NK and cause apoptosis in target cells [169]. In normal immune synapses the release of granzyme B is accompanied by perforin that forms a pore in a cell membrane and lets granzyme B in [170]. Thus, it was expected that the GzmB would kill target cells only in the presence of permeabilizing or endosmolytic substances, such as chloroquine [171], which is consistent with a natural way of the GzmB delivery into a cell. Nevertheless, GzmB and GzmB-based immunotoxins, used in a number of works without additional permeabilizing agents, exhibited a considerable antitumor efficiency [118]. The main argument against GzmB, apart from the lack of translocation signal, is the risk of injecting an active protease into the circulation, normally not found in this environment.

### 4.5. Enhanced Diffusion of Other Anticancer Drug

One more group to mention includes protein toxins that are not effective against cancer themselves, but can provide a synergistic effect with other anticancer drugs due to enhanced tumor or cell penetration. We have already mentioned junction opener and cell-penetrating peptides as means of drug delivery [56], but protein toxins can also contribute to drug diffusion enhancement, mainly cholesterol-dependent cytolysins (CDC) [172]. The Listerilysin O (LLO) produced by the bacterium *Listeria monocytogenes* is noticeable because of its reversible activation: this pH-sensitive protein acts as cytolysin in acidic environment of endosomes and lysosomes and is inactivated in extracellular media and cytoplasm mainly due to pH [173,174]. LLO in a form of recombinant targeted protein was shown to facilitate the action of gelonin-based targeted toxin in vitro and decrease IC_50_ by several orders of magnitude due to enhanced endosome release of the toxin [68]. LLO was also used in vitro for the cytoplasm delivery of liposome-encapsulated gelonin [85]. Another CDC protein, the Streptolysin-O (SLO) produced by *Streptococcus pyogenes* was shown to increase cytoplasmic delivery of various proteins including active domains of large clostridial toxins from *Clostridium difficile* B-toxin, *Clostridium sordelli* lethal toxin, and *Clostridium botulinum* C2 toxin [119]. SLO was also used in vitro to increase sensitivity of head and neck squamous cell carcinoma cells to Telomelysin (OBP-301), a telomerase-specific replication-competent adenovirus with a human telomerase reverse transcriptase (hTERT) promoter [120].

Enhanced tumor penetration can also be achieved due to the better blood supply. It was shown that botulinum neurotoxin A (BoNT-A) causes tumor blood vessels dilatation thus providing better tumor perfusion and oxygenation. The local intratumor administration of BoNT-A caused significant reoxygenation and reperfusion of tumors in vivo leading to a significant increase in the efficacy of X-ray radiotherapy and cyclophosphamide therapy at the time of maximal reoxygenation and reperfusion [175]. In another work the increase in the delivery of gemcitabine into tumors following treatment with BoNT-A was observed [176]. It is interesting, that BoNT alone did not alter apoptosis in tumor cells or induce any radio-sensitizing effect in vivo and the benefit from BoNT was directly related to a change in the tumor microenvironment [175].

## 5. Reducing Protein Toxins Side Toxicity

The protein toxins high toxicity is one of main advantages of these molecules but at the same time it increases the risk and severity of side effects. The side toxicity of a protein can be based on a direct cell killing and inflammation induction [177]. The most common side effects caused by DT, PE, and ricin include vascular leak syndrome, hepatotoxicity, and kidney damage [84,121,122]. In addition, Shiga toxin is notorious for its ability to cause hemolytic uremic syndrome (HUS), potentially leading to life-threatening complications [123,124]. The production of neutralizing antibodies can also serve as a cause on side effects due to anaphylaxis reactions. 

To date the number of strategies were developed to reduce protein drug off-target toxicity, the main tools are summarized in Table 2.

The natural tropism of a toxin can sometimes be used to target a tumor, as we have already discussed for anthrax toxin and Shiga toxin, but for the majority of protein toxins the natural tropism provides an off-target activity. To reduce the unwanted effects it is desirable to impair the targeting moieties. It was first implemented for the toxins consisting of targeting and effector modules, which predisposes them to be used in the truncated form. The truncated forms of protein toxins were used in the second generation of immunotoxins, which helped to reduce their in vivo side toxicity retaining their efficiency [16]. The targeted proteins with truncated toxins were first acquired with the use of DT and ricin [16], then the promising specific toxicity was proven for PE40, the engineered ETA [180,181]. Further miniaturization of PE led to the remarkable success in reducing both its immunogenicity and side toxicity. The PE-fused antibodies and other targeting proteins efficiently kill cancer cells in vitro [94,178,182] and reduce or stop the growth of tumors of various origin in vivo [22,94,183]. However, PE is notorious for its high immunogenicity: PE is a bacterial protein that can induce antibody responses and has a considerable side toxicity [184,185]. Although PE-based agents can be used successfully in combination with immunosuppressive chemotherapy [184] or in the treatment of hematologic malignances [186,187], the production of neutralizing antibodies reduces the efficiency of the PE-based therapy in patients with intact immune system and increases the probability of hypersensitivity reactions. The removal of domain II leads to a decrease in immunogenicity and, at the same time, reduces the protein degradation in the lysosomes. In addition, it helps to reduce off-target side toxicity in animal models [188]. Further investigation of PE helped to map the immunodominant epitopes of the catalytic domain and make them less visible to immune cells by deletions and point mutations [45]. The resulting toxin variants demonstrate high anti-cancer activity comparable to the activity of the initial variants of PE40 and PE38 and have decreased side toxicity and are less immunogenic [52,53,178]. 

For the anthrax toxin the introduction of point mutations impairing natural targets binding has proven to be effective: a double mutation in domain 4 of protective antigen (PA) led to the ablation of the protein native receptor-binding function. The resulting mPA fuse with EFG in a complex with LFN-DTA efficiently inhibited protein synthesis in EGFR-positive A431 cells in vitro (IC_50_ = 10 pM) not affecting the protein synthesis of CHO-K1 cells lacking EGFR. This variant was also used to target cells expressing HER2 [146,189], and both EGFR and carcinoembryonic antigen [190]. Still, the tumor-killing activity and side toxicity of these proteins in vivo are yet to be investigated. 

In case of glycoproteins, the oligosaccharides involved in off-target binding can be chemically removed. The ricin oligosaccharides bind to CD206 mannose receptor on macrophages [38,46], and interact with glycosylated IgA and IgM [47]. The circulation time and anti-tumor activity of ricin-based immunotoxins can be increased by chemical deglycosylation of the toxin [39,48], but unfortunately, these forms are more toxic to mice than the glycosylated ones [39]. 

Another promising strategy relies on tumor-specific activation of a toxin that requires proteolytic cleavage for toxin functioning. Several toxins, namely DT, PE, and ricin are digested in endosomes by furin protease thus releasing active protein fragments. By means of gene engineering the furin cleavage site can be replaced by the sequences recognized by the proteases that are upregulated in tumors. This strategy was realized for anthrax toxin protective antigen (PA): it was obtained in matrix metalloproteinase-dependent and urokinase plasminogen activator-dependent variants [138,139] which were selectively activated by tumor cells expressing respective proteases. The MMP-activated PA in combination with anthrax toxin lethal factor efficiently treated melanoma xenografts, and lung and colon carcinoma xenografts irrespective of the B-RAF status, targeting not only tumor cells, but also tumor vasculature [137]. This engineered toxin was less toxic than wild-type LT to mice because of the limited expression of MMPs by normal cells and also displayed lower immunogenicity compared with the wild-type toxin. The systemically administered toxin produced greater anti-tumor effects than wild-type LT toward human xenograft tumors. Both types of activated PA molecules were used to obtain dual-activity dependent delivery system based on PA variants that can only form octamers after activation by both of the tumor-selective proteases, uPa and MMPs. This complex agent completely stopped tumor growth in mice and its components were well tolerated in higher doses, than the wild-type PA and LT [140].

The most recent strategy for prevention of toxic agents intake by macrophages is based not on a toxin modifications, but on a transient reticuloengothelial (RES) cells inactivation. It can be achieved either by injection if blocking nanoparticles [179] or by enhanced clearance of erythrocytes caused by anti-erythrocyte antibodies [78]. These methods were successfully used to prolong nanotherapeutic agents circulation time and can be possibly applied for toxin-based therapy.

## 6. Conclusions

Cancer treatment has been revolutionized due to antigen-targeting drugs that specifically deliver a cytotoxic component to cancer cells, and advances in genetic engineering and biotechnology, making it possible to produce any fusion proteins needed. Potent cytotoxic components include enzymatically active protein toxins based on plant or bacterial toxins. Here, we have summarized several decades of research devoted to targeting internalizing receptors of cancer cells with chimeric therapeutic molecules. The targeting approach can also be applied to drug carriers such as liposomes, polymers, and nanoparticles. The design of complex targeted agents or several drug application regimens that allow achieving a synergistic effect is also a promising area of anticancer therapy. 

The use of several toxic mechanisms or several target molecules makes it possible to compensate for the deficiencies of effector molecules, increase their efficiency and avoid selection of resistant cells. The designed toxic proteins capable of ROS production and fused to UCNP or luciferase make it possible to overcome the shallow depth of excitation light penetration, thus providing a novel approach to PDT of deeply located tumors. 

Despite the numerous breakthrough solutions in cancer treatment, the problem is still far from being solved. It is worth mentioning that only two toxin-based molecules, namely Diphtheria toxin-based DAB_389_IL2 and DAB_389_IL3 [191,192], have been approved in late-stage clinical evaluation. Recently, a PE-based immunotoxin Moxetumomab Pasudotox (Lumoxiti), targeting CD22, has been approved for the treatment of patients with hairy cell leukemia [53]. In the future, new targeted therapies and combinations with increased selective anticancer activity and minimal side effects will be studied, which will increase the clinical efficacy of patients with various types of cancer.

## Figures and Tables

**Table 1:** Protein moieties commonly used in experimental anticancer therapy.

Mechanism of Action	Details	Examples	References
eEF2 inactivation	ADP-ribosylates elongation factor 2 (eEF2) and halt protein synthesis at the elongation step	Pseudomonas exotoxin A (PE, ETA)	[62,106]
		Diphtheria toxin (DT)	[12,83]
Ribosome inactivation	N-glycosidase depurinates a critical adenine in 28S rRNA, which results in the inability of the ribosome to bind elongation factor 2, thereby blocking protein translation	Ricin	[63,107,108]
		Shiga toxin (Stx)	[30]
		Abrin	[109,110,111]
RNA degradation	Nonspecific RNA cleavage blocks protein synthesis and leads to apoptosis	Barnase	[112,113]
		Binase	[114]
Cell signaling disruption	The cleavages of the MAP kinase family members leading to their inactivation; uncontrolled conversion of ATP to cAMP	Anthrax toxin	[115]
Photoinduced ROS production	The proteins absorb exciting light and produce reactive oxygen species	KillerRed	[116,117]
		miniSOG	[6]
Direct apoptosis induction	Effector caspases cleavage	Granzyme B	[118]
Enhanced diffusion of anticancer drug	Vascular network modulation	Botulinum neurotoxin	[57,58]
	Pore formation for better intracellular delivery	Listeriolysin O	[68,85]
		Streptolysin-O	[119,120]

**Table 2:** The strategies for reduction protein toxin side toxicity.

Strategy Used for Side Toxicity Reduction	Principle	References
Impairment of natural tropism	Removing the natural targeting domains of AB toxins	[35]
	Introduction of point mutations attenuating the target binding	[145]
Construction of miniaturized toxin variants	Deletion of protein parts not directly involved in toxin mechanism of action to reduce any non-specific interaction and immunogenicity	[52,53,178]
Tumor-specific activation of a toxin	The replacement of furin cleavage site to tumor-specific proteases cleavage sites (MMP, uPA)	[138,139,140]
RES cells inactivation	Macrophages blockade decreasing toxic nanoparticles uptake	[78,179]

## Abbreviations

DT	Diphtheria toxin
PE	*Pseudomonas aeruginosa* exotoxin A
RIT	Ribosome inactivating toxin
Stx	Shiga toxin
HER2	Human epidermal growth factor receptor 2
EpCAM	Epithelial cell adhesion molecule
FcRn	the neonatal immunoglobulin Fc receptor
MMP	Matrix metalloprotease
uPA	Urokinase plasminogen activator
